# Scanning Laser Optical Tomography Resolves Structural Plasticity during Regeneration in an Insect Brain

**DOI:** 10.1371/journal.pone.0041236

**Published:** 2012-07-19

**Authors:** René Eickhoff, Raoul-Amadeus Lorbeer, Hannah Scheiblich, Alexander Heisterkamp, Heiko Meyer, Michael Stern, Gerd Bicker

**Affiliations:** 1 Division of Cell Biology, University of Veterinary Medicine Hannover, Hannover, Germany; 2 Biomedical Optics Department, Laser Zentrum Hannover e.V., Hannover, Germany; 3 Institute of Applied Optics, Friedrich-Schiller-University Jena, Jena, Germany; 4 Institute of Quantum Optics, Leibniz University Hannover, Hannover, Germany; Aix Marseille University, France

## Abstract

**Background:**

Optical Projection Tomography (OPT) is a microscopic technique that generates three dimensional images from whole mount samples the size of which exceeds the maximum focal depth of confocal laser scanning microscopes. As an advancement of conventional emission-OPT, Scanning Laser Optical Tomography (SLOTy) allows simultaneous detection of fluorescence and absorbance with high sensitivity. In the present study, we employ SLOTy in a paradigm of brain plasticity in an insect model system.

**Methodology:**

We visualize and quantify volumetric changes in sensory information procession centers in the adult locust, *Locusta migratoria*. Olfactory receptor neurons, which project from the antenna into the brain, are axotomized by crushing the antennal nerve or ablating the entire antenna. We follow the resulting degeneration and regeneration in the olfactory centers (antennal lobes and mushroom bodies) by measuring their size in reconstructed SLOTy images with respect to the untreated control side. Within three weeks post treatment antennal lobes with ablated antennae lose as much as 60% of their initial volume. In contrast, antennal lobes with crushed antennal nerves initially shrink as well, but regain size back to normal within three weeks. The combined application of transmission-and fluorescence projections of Neurobiotin labeled axotomized fibers confirms that recovery of normal size is restored by regenerated afferents. Remarkably, SLOTy images reveal that degeneration of olfactory receptor axons has a trans-synaptic effect on second order brain centers and leads to size reduction of the mushroom body calyx.

**Conclusions:**

This study demonstrates that SLOTy is a suitable method for rapid screening of volumetric plasticity in insect brains and suggests its application also to vertebrate preparations.

## Introduction

Central nervous systems are able to change their physical structure as a consequence of disruption and restoration of sensory input. Insects are useful model organisms for studying such structural plasticity of the nervous system. It is well established that insect brains retain plastic capacities during adult live associated with changes in neuropil volume and alterations in synaptic circuitry [1], [2]. These phenomena have been implicated in age-dependent, experience-dependent, and behavioral-coupled reorganization of the nervous system [3]–[6]. Furthermore, plastic changes in the nervous system occur after injury. Whereas the regenerative capacity of the central nervous system (CNS) in higher vertebrates is rather low [7], insects are capable of neuronal regeneration both in the peripheral nervous system (PNS) and the CNS [8]–[12]. Thus, basal mechanisms of neuronal regeneration can be studied in insect models without the need to overcome inhibitory extrinsic factors. We have recently introduced the olfactory pathway of the locust *Locusta migratoria* as a model system for neural regeneration demonstrating that axonal regeneration in this system is possible and beyond that, fast and precise [13].

The primary olfactory organs of insects are the antennae. In the locust, each antenna houses 50.000 olfactory receptor neurons whose axons project into the antennal lobe, the primary olfactory center [14]. Intriguingly, the synaptic organization of the insect antennal lobe shares conspicuous similarities with the olfactory bulb in vertebrates [15]. Similar to the vertebrate olfactory bulb, in the antennal lobe olfactory receptor neurons synapse with local interneurons and olfactory receptor neurons (vertebrates: mitral-and tufted cells) in spherical neuropils called glomeruli. In the locust, olfactory receptor neurons converge onto local interneurons and 830 olfactory projection neurons in multiple microglomeruli [16], [17]. The axons of the olfactory projection neurons leave the antennal lobe via the antennocerebral tract and project toward second-order neuropils, the lateral horn and the mushroom body [16], [18]. In the dendritic compartment of the mushroom body, the calyx, projection neurons diverge to ca. 50.000 densely packed mushroom body intrinsic neurons, the Kenyon cells. Due to their prominent role in multimodal sensory integration and olfactory memory formation, mushroom bodies have been compared to higher vertebrate brain centers such as the cerebellum and the hippocampus [19].

The analysis of structural plasticity requires the 3D representation of the biological object. Thanks to vast advances in imaging technology, the last decades have provided us with a variety of tools that allow for generating three-dimensional images of organs and whole mount preparations [20]. 3D reconstruction of optical sections with confocal laser scanning microscopy (CLSM) achieves particularly high spatial resolution [20]. Moreover, this method has the advantage that the tissue has not to be physically cut, minimizing distortions of the tissue due to sectioning-and embedding procedures. However, this method also bears several disadvantages. For instance CLSM is mainly applied to fluorescence labeled tissue. Satisfactory results therefore depend on proper tissue penetration of the antibodies and/or fluorescent dyes and demand long incubation periods. Moreover, in CLSM maximum sample size is limited due to the low working distance of the commonly used objectives (∼400–500 µm) and limited optical penetration depth. Hence, scans of whole mount preparations are often difficult [21] or not possible at all. These reasons render CLSM-based 3D reconstructions and imaging with selective plane illumination [22] as unpractical for routine and high throughput applications. Optical Projection Tomography (OPT) is a relatively novel technique that has been proven to reliably generate 3D images of specimens with sizes up to several millimeters [20], [23]. In brief, transmitted, scattered, or fluorescent light images of a rotating sample are collected from multiple viewing angles. These images are further reconstructed into tomographic sections covering the entire depth of the preparation similar to x-ray projections in computed tomography (CT) [24]. Analogous to absorption, emitted light can be used to create image contrast (eOPT). Here we use a laser based OPT setup, referred to as Scanning Laser Optical Tomography (SLOTy), that has recently been introduced by Lorbeer et al. [24] to optimize efficiency significantly when compared to conventional eOPT. Our setup allows detecting transmission and fluorescence at the same time. Hence, both datasets can be superimposed 1∶1 to form one image comprising the transmission projection and the fluorescence signals.

This study addressed whether SLOTy is a suitable method to visualize and quantify size changes in the olfactory centers of the locust after unilateral deafferentation. In addition, we asked whether neuronal degeneration of the antennal lobe is transmitted trans-synaptically and thus affects the size of the mushroom body calyx.

## Materials and Methods

### Animals

All experiments were performed on adult *Locusta migratoria* of both sexes, aged max. one day after imaginal moult. Animals were bred in crowded colonies reared under standard conditions under a 12/12 hour photoperiod. All experiments were performed according to the German laws for animal care (Deutsches Tierschutzgesetz). All chemicals were purchased from Sigma (St. Louis, MO), unless stated otherwise.

### Deafferentation

Antennal nerve crush was performed on cold-anesthetized animals using specially fashioned forceps with narrow but blunted shanks. The scapus of the right antenna was crushed for 13 s. The other antenna was left intact, serving as an internal reference. In a second approach, the flagellum of the right antenna was ablated with fine scissors to follow maximum degeneration over the experimental period. After treatment, animals were kept under standard rearing conditions until they were subjected to SLOTy at different time points (0, 7, 14, and 21 days) after nerve crush/ablation in batches of five (ablation) or ten (nerve crush) specimens. In nerve crush experiments, only animals without obvious damage to either antenna were included.

### Dissection

For dissection, fixation, and optical clearing of locust brains we adapted a protocol described by Ott [25] to minimize anisotropic shrinking of the tissue. In brief, brains were dissected in HEPES buffered saline (HBS; 150 mM NaCl; 5 mM KCl; 5 mM CaCl_2_; 25 mM sucrose; 10 mM HEPES, pH 7.4) and fixed in zinc-formaldehyde (ZnFA; 18.4 mM ZnCl_2_; 135 mM NaCl; 35 mM sucrose, and 1% formaldehyde) for 20 hours at room temperature. After washing in HBS, preparations were cleared in a graded series of glycerol (1%, 2%, and 4%, 2 h each; 8%, 15%, 30%, 50%, 60%, 70%, 80%, and 87%, 1 h each). Glycerol was diluted in 0.1 M Tris/HCl, pH 7.8. Dimethylsulfoxide (DMSO) was added to a final concentration of 1%. For SLOTy, cleared preparations were transferred into glass capillaries in 87% glycerol.

### Anterograde Labeling

For anterograde labeling of antennal nerves animals were cold anesthetized on ice and then immobilized with plasticine on a petri dish. Antennae were cut at the tenth flagellar annulus, and approximately 1 mm of the distal ends of both antennal nerves was dissected free under locust saline. Nerve ends were placed in a reservoir with approximately 10–20 µl of 5% Neurobiotin (Vector Laboratories, Burlingame, CA) in phosphate-buffered saline (PBS; 10 mM sodium phosphate, 150 mM NaCl, pH 7.4) and sealed in petroleum jelly. Preparations were incubated overnight at 4°C. Brains were dissected in HBS and fixed in ZnFA for 20 hours at room temperature. Preparations were permeabilized for 2–4 hours in 0.3% saponin in HBS with Triton X-100 (HBS-T), washed in HBS-T and incubated in Cy3-conjugated streptavidin (1∶250) overnight at 4°C. After final washing steps in HBS, brains were subjected to clearing procedure as described above.

### Scanning Laser Optical Tomography Setup

**Figure 1:**
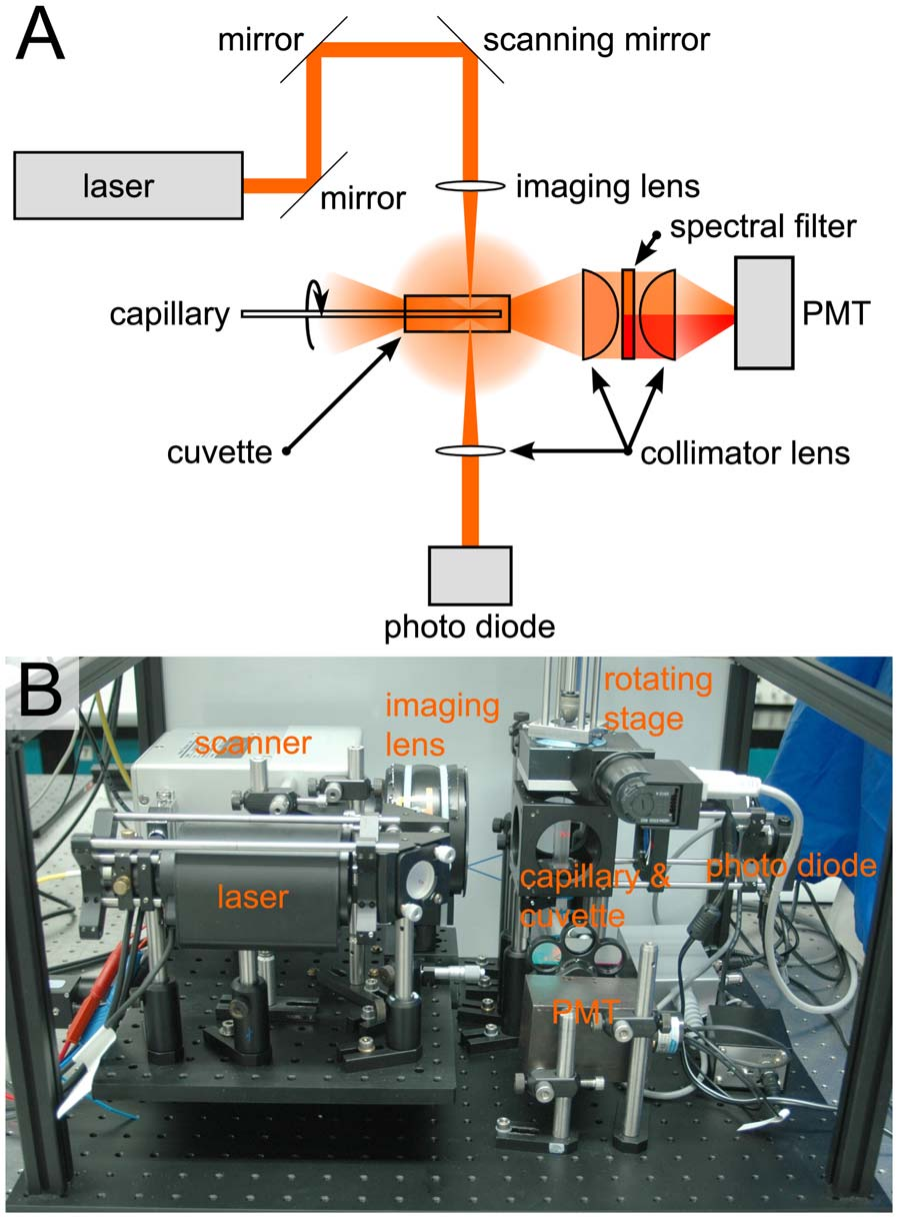
Scanning Laser Optical Tomography setup. Schematic drawing (A) and photograph (B) of the Scanning Laser Optical Tomograph. A laser beam is directed via two mirrors onto an x-y-galvanometer-scanning system. The system is placed in the back focal plane of the imaging lens, which produces a weakly focused beam in the imaging volume. Transmitted light collected with a collimating lens onto a photo diode. Scattered and fluorescent light is collimated with a lens at the bottom of the cuvette. A spectral filter suppresses the scattered light for fluorescence detection (indicated in red; in A) or the fluorescent light for scattered light detection (indicated in orange; in A). The filtered light is then collimated onto a photomultiplier tube (PMT). A capillary containing the sample is rotated within the cuvette which is filled with glycerol.

The Scanning Laser Optical Tomograph is shown in Figure 1. In principle, the Tomograph is an X-ray computed tomography (CT) scanner working with visible light. While today fan beam scanners are commonly in use, G. N. Hounsfield [26] used a sequential acquiring single beam CT scanner which is equivalent to a SLOTy setup. SLOTy makes use of a weakly focused laser beam passing through the sample at various lateral scanning positions with a Rayleigh range covering the full thickness of a sample in z-direction. The fluorescence, scattering, or absorption is measured for each lateral position of the laser beam. After a complete two dimensional scan one single projection of the object has been accomplished. 500 single projections from equidistant viewing angles are acquired resulting in one full revolution of the sample. The overall acquisition time per sample is 22 minutes. Afterwards, the acquired dataset is reconstructed like in X-Ray CT by an inverse Radon transformation. The computation takes place on consumer PC hardware. To cope with the amount of data, the reconstruction algorithms have been implemented on graphics hardware similar to the approach of C. Vinegoni et al. [27].

For this study three imaging modalities had to be available:

The first one is the detection of transmitted light. A photodiode with a collimating lens was used to collect the attenuated laser light passing through the sample. The only other optical technique known so far of being capable to acquire three dimensional transmission data is OPT [20].

The second modality needs the detection of scattered light. Therefore light scattered by the sample at each scanning position, which travels to the bottom of the cuvette is collected by a lens. A spectral filter (Semrock, Rochester, NY, 624/40) transmits the laser wavelength of 635 nm (Conrad Electronic, Hirschau, Germany, red laser diode) and excludes stray or fluorescent light. The transmitted light is then collimated and detected by a photomultiplier tube (PMT; Hamamatsu Photonics, Hamamatsu City, Japan, R3896).

The third modality is the detection of fluorescent light of the fluorophore Cy3. In this case the setup is identical to the detection of scattered light, while the laser wavelength is shifted to 532 nm (Insaneware Deluxe, Gladbeck, Germany, green laser module) and therefore blocked out by the spectral filter.

The benefit of this arrangement lies in the simultaneous acquisition of three dimensional transmission data together with either the second (scattering) or the third (fluorescence) imaging modality. Therefore, the acquired information can be superimposed without any bias or scaling errors. Additionally, the dynamic range of the fluorescence images is two orders of magnitude higher than with OPT.

To account for optical deficiencies of the sample, the sample has to be chemically cleared (see dissection) and then mounted and placed within an index matching fluid. In this case the cuvette was filled with 99% glycerol and the capillary with 87% glycerol (see dissection).

### Image Acquisition and Data Analysis

Image processing, including contrast and brightness adjustment, and movie preparations were carried out using NIH ImageJ (version 1.45, W. S. Rasband, ImageJ, US National Institutes of Health, Bethesda, MD, http://rsb.info.nih.gov/ij/). In some cases contrast was enhanced using the “Enhance Contrast” tool and the “Unsharp Mask” filter. Figures were arranged in Adobe Photoshop.

### Statistics

Data are presented as mean ± SEM. Datasets were tested for Gaussian distribution with Kolmogorov-Smirnov test. Normally distributed data were tested with one-way ANOVA followed by Tukey’s multiple comparison tests. In cases where datasets were not normally distributed, Mann Whitney U-test or Kruskal-Wallis test followed by Dunn’s post hoc test was applied. Differences were considered significant if p<0.05. *p<0.05, **p<0.01, ***p<0.001. Statistic analyses and figures were prepared with GraphPad Prism.

## Results

### Optical Tomography of an Insect Brain

**Figure 2:**
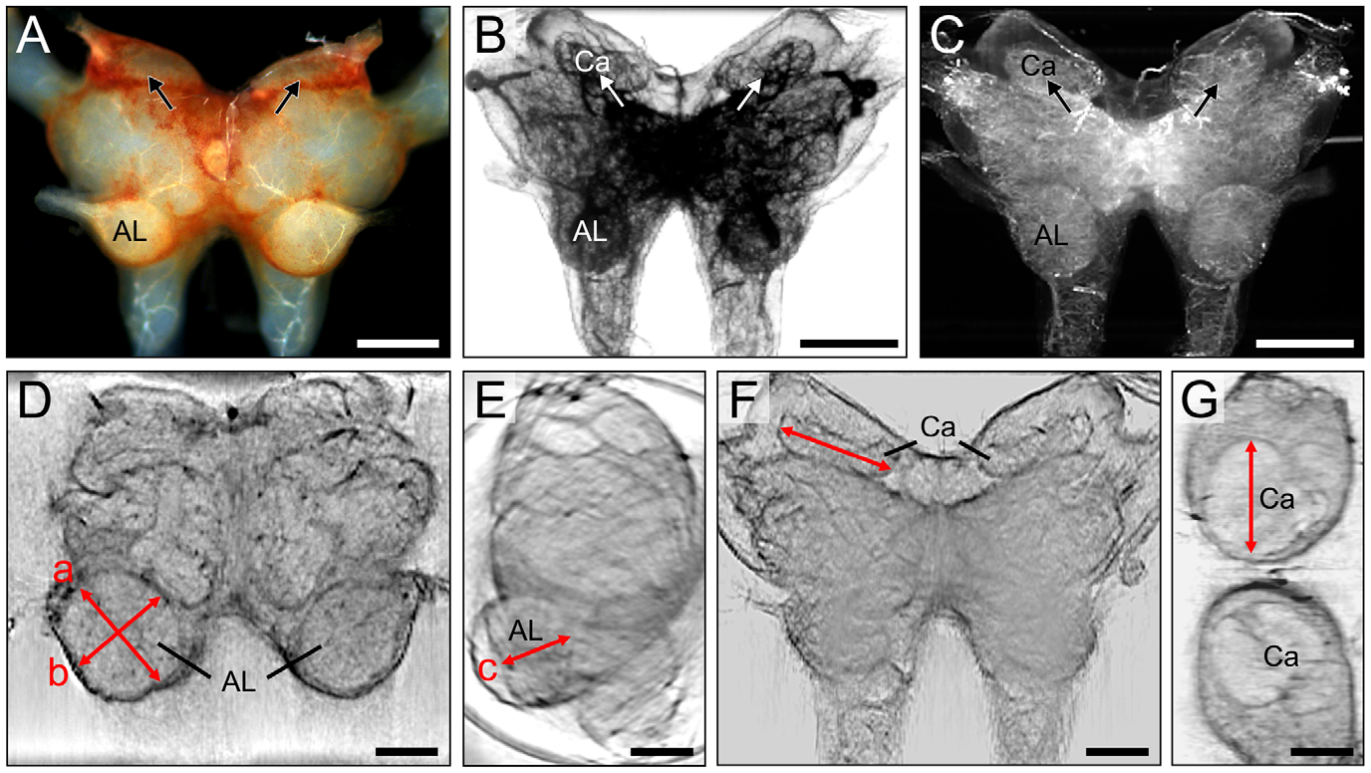
Evaluation of size changes of the antennal lobe and mushroom body with Scanning Laser Optical Tomography (SLOTy). A. Frontal view of the untreated locust brain (exclusive optic lobes) under reflected light. The mushroom body calyces are hardly visible (arrows). B,C. Raw data of 3D transmission-(B) and scattered light (C) projections of the locust brain. Brains were cleared with glycerol. Note that mushroom body calyces are clearly visible (arrows in B,C). D–G. Reconstructed 2D optical sections of transmission projections. D. Frontal section depicting left (untreated side) and right (ablated side) antennal lobe 7 days after ablation of the right antenna. Note that the right (deafferented) antennal lobe is smaller than the left (untreated) antennal lobe. E. Side view of brain with left antennal lobe. Antennal lobe size was approximated as an ellipsoidal volume V = π/6abc (D,E). F,G. Frontal section (F) and horizontal section (G) depicting mushroom body calyces. Calyx width was measured in frontal sections (red line in F). AL, antennal lobe; Ca, calyx. Scale bars  = 400 µm in A–C; 200 µm in D–G.

Optical tomography of the nervous system depends crucially on the transparency of the tissue while retaining sufficient contrast for imaging the various brain compartments. In pilot experiments we tested different concentrations of glycerol as optical clearing agent for the locust brain. A concentration of 87% glycerol yielded appropriate contrast to resolve the overall anatomy of the olfactory neuropils and was further used in all preparations. A total of 63 locust brains were subjected to SLOTy. To create tomographic slices of the central brain, samples were introduced into a glass capillary and 500 images were scanned at equally spaced angles over 360° (equivalent to consecutive intervals of 0.7°) using transmitted or scattered light (Figure 2). Whereas the mushroom body calyces are hardly visible in uncleared brains (Figure 2A), both transmission and scattered light projections of cleared brains revealed the shape of the calyx neuropil (Figure 2B,C). Due to their exposed position, antennal lobes are already visible in uncleared brains, however, only tomographic slices of cleared brains revealed the geometry of their internal neuropils. These reconstructed images enabled us to distinguish the spheroid shape of the antennal lobe neuropil (Figure 2, double arrows a,b,c in Figure 2D,E) from the surrounding rind of cell somata. Because signal overlap with the subjacent tissue did not provide sufficient contrast in the raw-projections at some viewing angles, we used optical sections of the reconstructed data to evaluate the size of the analyzed neuropil. For this purpose transmission projections provided the best results (Figure 2D–G; Movie S1). Re-slicing of reconstructed 3D datasets allowed to create XY images in multiple planes, such as frontal sections (Figure 2D,F) and horizontal sections (Figure 2G). Optical sections resolved not only the neuropils mediating chemosensory information processing including the antennal lobe, mushroom body calyx, pedunculus and mushroom body lobes, but also other brain centers, such as the central body (CB in Movie S1).

### Volume-size Changes of the Antennal Lobe after Sensory Deafferentation

**Figure 3:**
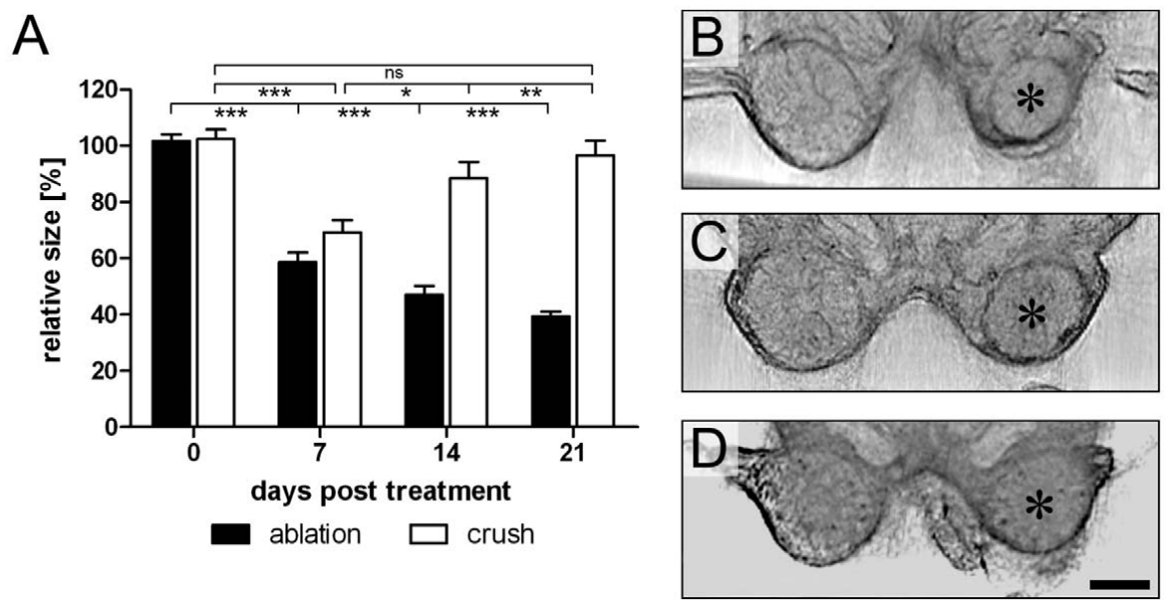
Changes of antennal lobe size after unilateral deafferentation via antennal ablation and nerve crush. A. Relative size of right (treated) antennal lobe expressed as percentage of left (control) side. Datasets are based on estimated neuropil volume. Each bar represents the mean value of 5–10 preparations. Error bars indicate SEM. Levels of significance are indicated *p<0.05, **p<0.01, ***p<0.001. B–D. Reconstructed optical sections of antennal lobes after unilateral deafferentation. Asterisks indicate treated side. B. 21 days after ablation of right antenna. Size of right antennal lobe is reduced by ca. 60%. C. 7 days after nerve crush of right antenna. Size of right antennal lobe is significantly reduced compared to left (control) side. D. 21 days after nerve crush. Right antennal lobe has regained size almost back to normal. ns, not significant. Scale bar  = 200 µm in D (applies to B–D).

Antennal lobes shrink after crushing the antennal nerve and regain size almost back to normal within 14 days [13]. Here we use SLOTy to follow size changes of the antennal lobe after unilateral nerve crush or antennal ablation over a period of 21 days. We examined 40 experimental animals with the right antenna crushed and 20 animals with the right antenna ablated. Because neuropil size varied considerably (ca. 20%) among individuals (mean volume control side: 3.4×10^7^ µm^3^±0.7×10^7^ µm^3^), sizes of affected neuropils were normalized to percent of the untreated (control) side. In both groups antennal lobes were on average of almost equal size at zero days post treatment (treated vs. control side: 100%±11%). After 7 days antennal lobes of animals shrunk to ca. 60% of the control side when the antenna had been ablated and to 70% when the antennal nerve had been crushed (Figure 3A,C; p<0.001, one-way ANOVA, with Tukey’s multi comparison test). In the ablated group antennal lobes continuously degenerated and lost as much as 60% of their initial volume within 21 days (Figure 3A,B; p<0.001, one-way ANOVA, with Tukey’s multi comparison test). In contrast, antennal lobes of animals with crushed antennal nerves significantly regained size from 7 days onward and within 21 days regained size back to 96,6% of the control side (Figure 3A,D; p<0.01, one-way ANOVA, with Tukey’s multi comparison test).

### Size Changes of the Mushroom Body after Sensory Deafferentation

**Figure 4:**
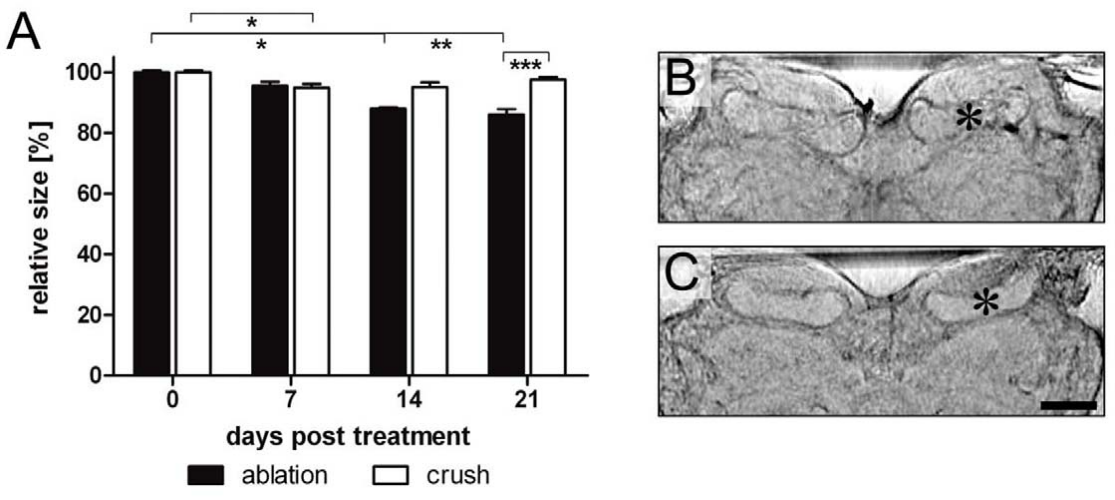
Changes of mushroom body size after unilateral deafferentation of the antennal lobe via antennal ablation and nerve crush. A. Relative size of right mushroom body calyx (treated side) expressed as percentage of left (control) side. Each bar represents the mean value of 5–10 preparations. Error bars indicate SEM. Levels of significance are indicated *p<0.05, **p<0.01. B,C. Reconstructed optical sections of mushroom body calyces after unilateral deafferentation of the right antennal lobe. Asterisks indicate treated side. B. 21 days after ablation of right antenna. Size of right calyx is reduced by ca. 10%. C. 7 days after nerve crush of right antenna. Size of right calyx is significantly reduced compared to left (control) side. Scale bar  = 200 µm in C (applies to B,C).

Next we examined whether deafferentation of the sensory input affected the size of the second order olfactory neuropil, the mushroom body. We focused our study on the mushroom body calyx, the compartment where afferent olfactory projection neurons synapse with mushroom body intrinsic neurons. Since the overall shape of the mushroom body calyx does not correspond to a simple geometric body like the antennal lobe, we restricted the analysis to measuring the calyx diameter (see Figure 2F,G). Values of calyx diameter did not obviously differ between measurements in frontal sections and horizontal sections. Hence, we analyzed only data collected in frontal sections. Similar to antennal lobe volume, calyx diameter varied (6.97%) among individuals (mean diameter control side: 481.37 µm±33.56 µm). No differences in calyx diameter between treated and control side could be observed at zero days post treatment (treated vs. control side: 99.94%±1.62%). In animals with ablated antenna, the ipsilateral calyx shrunk to 88% of the control side within 14 days (Figure 4A; p<0.05, Kruskal-Wallis test with Dunn’s post hoc test) and to 86% within 21 days (Figure 4A,B; p<0.05, Kruskal-Wallis test with Dunn’s post hoc test). In animals with antennal nerve crush, a slight but significant decline in calyx diameter (95% of the control) could be detected after 7 days (Figure 4A,C; p<0.05, Kruskal-Wallis test with Dunn’s post hoc test). No significant size differences to controls could be resolved after 14 and 21 days, suggesting regenerative processes within the olfactory pathway at later stages. Even though an increase in calyx diameter from 7 days onward could not be statistically confirmed, by the end of the experimental period calyces of animals with crushed antenna were profoundly larger than calyces of animals with ablated antenna (p<0.001, Mann-Whitney U-Test, two-tailed).

### Anterograde Labeling

**Figure 5:**
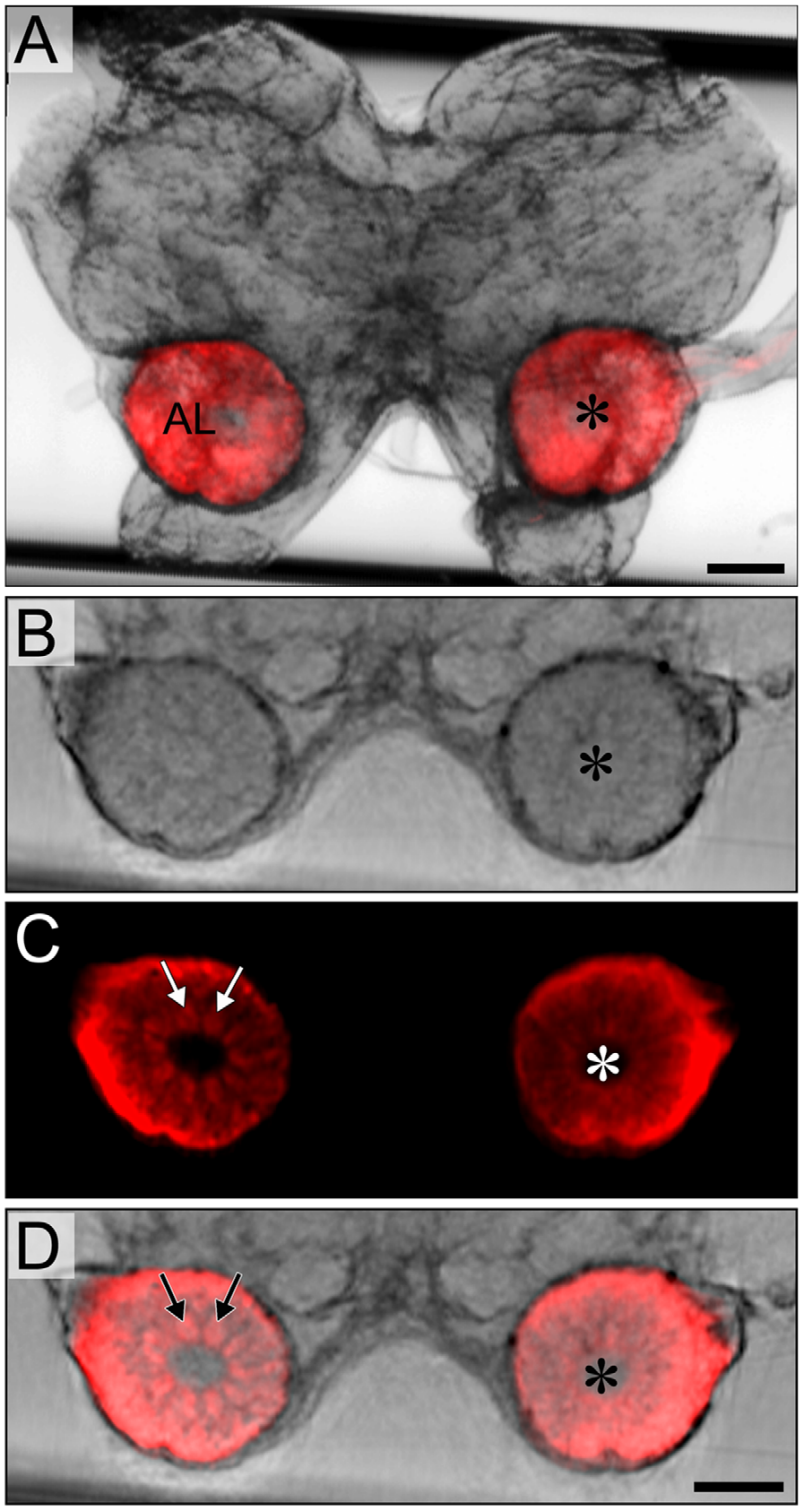
Superimposed transmission and fluorescence projections of a locust midbrain (exclusive optic lobes) 14 days after crush of the right antenna. Antennal lobes are labeled with Neurobiotin. Asterisks indicate treated side. No obvious differences in antennal lobe size are visible. A. Raw data of superimposed transmission-and emission projections. B–D. Reconstructed optical sections of antennal lobes. B. Transmission. C. Emission. D. Merged. Note that Neurobiotin labels distinct glomeruli in the left (control side; arrows in C,D) but not the right (treated side) antennal lobe. AL, antennal lobe. Scale bars  = 200 µm (scale bar in D applies to B–D).

To visualize regenerating sensory fibers in the antennal lobe we anterogradely labeled antennal nerves with Neurobiotin 14 days after nerve crush. In control experiments, absent fluorescence in antennal lobes immediately after nerve crush confirmed that the crush procedure was successful and that in later stages Neurobiotin exclusively labeled regenerated fibers. High contrast fluorescence signals from Neurobiotin-labeled antennal nerves could be collected with SLOTy and simultaneously superimposed with transmission projections (Figure 5; Movie S2). Similar to transmission images this method resolved no obvious differences in neuropil volume 14 days after nerve crush, indicating axonal regeneration of antennal fibers. The combined application of transmission-and fluorescence projections thus confirmed that recovery of normal size in deafferented antennal lobes was indeed restored by regenerated sensory fibers. Again, reconstructed 2D sections were used to visualize the analysed structure (see Movie S3). These images illustrate the glomerular organization of the antennal lobe. As shown in Figure 5C,D, Neurobiotin stained glomeruli in the left (untreated) but not the right (crushed) antennal lobe, suggesting that at this stage the full complement of synaptic connections between regenerated afferents and postsynaptic neurons had not yet been established.

## Discussion

Since its introduction by Sharpe et al. [28], Optical Projection Tomography (OPT) has been applied to a variety of samples including human embryos, whole mount Drosophila preparations, and even plant tissue [29]–[31]. Here, we adopted a novel OPT-technique, referred to as Scanning Laser Optical Tomography (SLOTy) [24], to follow structural changes in the brain of the model insect Locusta migratoria. Primary olfactory centers of the locust have the capacity to structurally regenerate upon sensory deafferentation [13]. In this study we showed that SLOTy can serve as a suitable method to rapidly illustrate and quantify neuronal plasticity of locust olfactory neuropils while maintaining the three dimensional structure of the brain. Using transmission projections we observed that deafferentation of the antennal lobe has striking effects on second order olfactory neuropils, the mushroom bodies. A major advantage of our setup is the potential to simultaneously detect absorption and fluorescence. By this means we resolved the 3D representation of fluorescent labeled regenerating axons within the antennal lobe neuropil.

### Advantages and Technical Limitations

OPT and SLOTy are optimized to acquire 3D images of specimens that are too big for Confocal Laser Scanning Microscopy (CLSM) [20]. Due to the low NA objectives needed to scan the whole mount sample, the spatial resolution obtained with SLOTy is lower compared to CLSM. However, SLOTy resolved immunofluorescent staining of identified neurons in the larval brains of the comparatively large locust and the smaller fruit fly at a cellular resolution [24]. In our present investigation we used SLOTy to illustrate the neuropilar organization of the locust brain and to reliably resolve neuronal plasticity on the anatomical level. In combination with fluorescence, structures as small as antennal lobe microglomeruli could be detected (Figure 5). Since SLOTy preserves the overall condition of the tissue, samples can first be scanned with SLOTy to obtain the 3D representation of the analysed structure and subsequently be processed for CLSM if a more detailed view is needed. The advantage of SLOTy over CLSM is the low time duration that is needed to create the high contrast image as well as the two additional imaging modalities of transmitted and scattered light. SLOTy could thus facilitate high throughput screenings of mutations affecting neuroanatomical topography [32] or the assessment of developmental neurotoxicity [33]. To identify genes regulating developmental processes, SLOTy could also advance the acquisition of digital 3 D images of gene expression patterns [34], [35] in whole mount embryos.

### Structural Plasticity in the Insect Brain

Due to the variability among individual specimens, neuropil size has been normalized to percent of the control side. These variations are, however, in the range of what has been described for the related species Schistocerca gregaria using CLSM [36]. The data we collected with SLOTy coincide well to what we have described with conventional microscopy [13]. Our experiments clearly confirm the regenerative potential of the locust antennal afferents. Because tomographic sections allowed approximating the antennal lobe volume, the information content was further improved.

It is likely that antennal lobe volume reduction is caused mainly by degeneration of the distal segments of olfactory afferents and that local and projection neurons remained unscathed [13]. Moreover, Neurobiotin labeling of axotomized olfactory fibers could clearly ascribe the recovery of antennal lobe volume to regenerated axons. Intriguingly, regenerating olfactory receptor axons express embryonic cell surface molecules indicative of reacquisition of a developmental mode [37], [13]. The observed time course of degeneration and subsequent regeneration is comparable to what has been shown in other insect sensory systems such as deafferented cercal fibers in the cockroach and auditory afferents in the grasshopper [38]–[40].

A novel finding of our analysis is that sensory deprivation of the antennal lobes also affects second order integration centers such as the mushroom bodies. Compared with the antennal lobe, the size reduction of the mushroom body calyx was apparently lower (ca. 14% reduction 21 days after ablation). However, for reasons of rapid screening we refer to calyx diameter and not volume. Hence, the actual magnitude of calyx volume reduction is much higher. The cellular mechanisms that underlie the volumetric changes remain to be elucidated in future analyses. Recently, Kremer et al. [2] have reported that in Drosophila reducing the firing rate of a small number of defined projection neurons leads to an increase in microglomeruli number and size in the calyx presumably compensating for the reduced presynaptic activity. A striking example of adult brain plasticity is the topographical reorganization of the primate somatosensory cortex following peripheral nerve damage [41]. Remarkably, the ablation of antennal sensory afferents reduced the size of the calyx region, emphasizing that the mushroom bodies of the adult locust are also capable of structural modifications in response to input deprivation.

### Conclusions

Using an insect regeneration paradigm we show that Scanning Laser Optical Tomography (SLOTy) is a suitable method for rapid screening of structural plasticity. This novel microscopic method allows for combining three dimensional transmission projections with fluorescence signals. SLOTy is perfectly suited to generate 3 D images of gene expression patterns in whole mount preparations of genetic model organisms such as Drosophila, chick embryo, zebrafish or the mouse brain.

## Supporting Information

Movie S1 **Reconstructed transmission images of a locust brain.** The Movie shows a sequence of reconstructed optical sections of transmission projections of a locust midbrain (exclusive optic lobes) 7 days after ablation of the right antenna. AL, antennal lobe; Ca, mushroom body calyx; CB, central body.
(AVI)

Movie S2 **Superimposed transmission and fluorescence projection of a locust brain.** The Movie shows the raw data of superimposed transmission and fluorescence projections of a locust midbrain (exclusive optic lobes) 14 days after crush of the right antenna. Antennal lobes are labeled with Neurobiotin.
(AVI)

Movie S3 **Reconstructed transmission and fluorescence images of a locust brain.** The Movie shows a sequence of reconstructed optical sections of superimposed transmission and fluorescence projections of a locust midbrain (exclusive optic lobes) 14 days after crush of the right antenna. Antennal lobes are labeled with Neurobiotin.
(AVI)
